# The effects of glycemic control on seizures and seizure-induced excitotoxic cell death

**DOI:** 10.1186/1471-2202-13-94

**Published:** 2012-08-06

**Authors:** Paula Elyse Schauwecker

**Affiliations:** 1 Department of Cell and Neurobiology, USC Keck School of Medicine, 1333 San Pablo Street, BMT 403, Los Angeles, CA, 90089-9112, USA

**Keywords:** Kainic acid, Excitotoxicity, Glucose, Epileptic seizures, Hypoglycemia, Hyperglycemia, Hippocampus, Mouse strain

## Abstract

**Background:**

Epilepsy is the most common neurological disorder after stroke, affecting more than 50 million persons worldwide. Metabolic disturbances are often associated with epileptic seizures, but the pathogenesis of this relationship is poorly understood. It is known that seizures result in altered glucose metabolism, the reduction of intracellular energy metabolites such as ATP, ADP and phosphocreatine and the accumulation of metabolic intermediates, such as lactate and adenosine. In particular, it has been suggested that the duration and extent of glucose dysregulation may be a predictor of the pathological outcome of status. However, little is known about neither the effects of glycemic control on brain metabolism nor the effects of managing systemic glucose concentrations in epilepsy.

**Results:**

In this study, we examined glycemic modulation of kainate-induced seizure sensitivity and its neuropathological consequences. To investigate the relationship between glycemic modulation, seizure susceptibility and its neuropathological consequences, C57BL/6 mice (excitotoxin cell death resistant) were subjected to hypoglycemia or hyperglycemia, followed by systemic administration of kainic acid to induce seizures. Glycemic modulation resulted in minimal consequences with regard to seizure severity but increased hippocampal pathology, irrespective of whether mice were hypoglycemic or hyperglycemic prior to kainate administration. Moreover, we found that exogenous administration of glucose following kainic acid seizures significantly reduced the extent of hippocampal pathology in FVB/N mice (excitotoxin cell death susceptible) following systemic administration of kainic acid.

**Conclusion:**

These findings demonstrate that modulation of the glycemic index can modify the outcome of brain injury in the kainate model of seizure induction. Moreover, modulation of the glycemic index through glucose rescue greatly diminishes the extent of seizure-induced cell death following kainate administration. Our data support the hypothesis that deficient insulin signaling may represent a critical contributing factor in the susceptibility to seizure-induced cell death and this may be an important therapeutic target.

## Background

Epilepsy is the most prevalent chronic neurologic disorder affecting over 3 million Americans of all ages [1,2] and is frequently refractory to current medical treatments [3]. Temporal lobe epilepsy (TLE), the most common form of epilepsy, produces a state of chronic neuronal hyperexcitability and hypersynchrony that is manifested as recurrent unprovoked partial seizures [4]. Hippocampal sclerosis, a common feature of TLE [5,6], is characterized by severe segmental neuronal loss in CA1, CA3, and the hilar region and is accompanied by pronounced astrogliosis [7]. Although the evidence of brain damage in humans, as a result of convulsive status epilepticus (SE), has been difficult to define or quantify, the marked variability in susceptibility to seizure-induced cell damage has been attributed to differences in the underlying pathology, age, and seizure type and duration [8-11]. Regardless, the molecular mechanisms involved in the pathogenesis of hippocampal sclerosis remain highly obscure. Thus, insight into these mechanisms is essential for the development of new neuroprotective drugs as, at present, no effective post-seizure treatment exists to prevent this brain injury.

Many of the pathophysiological consequences of human TLE (e.g. hippocampal sclerosis, mossy fiber sprouting, spontaneous seizures) are faithfully reproduced in the kainic acid (KA) chemoconvulsant rodent model of epilepsy [11-16]. Kainic acid, a potent agonist of the α-amino-3-hydroxy-5-methyl-4-isoxazoleproprionic acid/kainate class of glutamate receptors, is a powerful excitant and excitotoxin, which when injected directly into the brain or systemically induces a characterized pattern of persistent seizure activity [17], activates ionotropic glutamate receptors, and selectively induces excitotoxic cell death in postsynaptic neurons in the CA3 and CA1 hippocampal subfields and within the dentate hilus, while sparing neurons in the dentate granule cell layer [18-21]. Thus, KA administration has been widely used as a model to study excitotoxicity and seizure-related neurologic diseases [17,22].

While administration of kainic acid to rodents results in acute induction of seizures and subsequent neuronal damage, inbred mouse strains significantly differ in their pattern of hippocampal neurodegeneration in the KA model of TLE [23-29]. Interestingly, the duration or severity of seizure activity in response to KA is not predictive of subsequent hippocampal cell death. Although C57BL/6 (B6) and FVB/N (FVB) mouse strains exhibit comparable seizure activity following systemic administration of kainic acid (KA), C57BL/6 mice show essentially no hippocampal cell death. Those mice susceptible to KA administration show an excitotoxic response similar to what has been described in rats [18,30-32].

Metabolic disturbances are often associated with epileptic seizures, but the pathogenesis of this relationship is poorly understood. The importance of glucose balance has been identified in studies demonstrating that epileptic seizures can be exacerbated under conditions of hyper- or hypoglycemia [33-35]. Studies of type I and type II diabetic subjects have found that diabetes-related seizures usually improve with control of glycemic status [36]. In particular, the treatment of choice for hyperglycemia-related seizures is glycemic control, and seizures are usually resistant to antiepileptic drugs if blood glucose is not brought under control [37]. At present, the mechanisms underlying glucose regulation and altered neuronal excitability remain incompletely understood [38]. Nevertheless, despite the reported association between blood glucose levels and certain epilepsy syndromes [39], few studies to date have evaluated the efficacy of controlling blood glucose levels on seizure-induced neuronal injury.

In this study, we used mice that possess strain-specific gene products that can modify vulnerability to excitotoxin-induced cell death [27]. We were interested in identifying the relationship between glycemic index and susceptibility to seizure-induced excitotoxic cell death and establishing whether modulation of glycemic index could modify the susceptibility to epilepsy. As a first step, we wanted to determine if glucose administration following kainate-induced SE could reduce seizure-induced cell death in mice susceptible to excitotoxin-induced cell loss (FVB/N). Secondly, we wanted to determine if modulation of the glycemic index, in models of hypoglycemia or hyperglycemia, could affect seizure susceptibility and its neuropathological consequences following kainate administration in a mouse strain previously found to be resistant to seizure-induced cell death (C57BL/6).

## Results

### Seizure effects following systemic administration of kainate

Kainic acid (KA) is a selective glutamate receptor agonist and potent neurotoxin that when injected systemically, produces epileptic behavior and subsequent neurodegeneration [21,22]. As previously reported [18,27,40], administration of KA caused characteristic sequential behavioral changes. Within 15 minutes after KA injection, all animals began to exhibit behavioral signs of convulsive seizures. Seizure behavior was characterized by forelimb clonus and hindlimb clonus within 20–30 minutes after injection. Within 40–45 min after injection, all mice exhibited continuous tonic-clonic seizures that lasted for 1–2 hr. As shown in Table 1, we did not observe any strain-dependent differences in the percentage of mice exhibiting status epilepticus (stage 5 seizures), latency to onset of severe (stage 4/5) seizures, or duration of severe (stage 4/5) seizures.

**Table 1:** Effect of kainic acid administration on seizure parameters in normoglycemic, hypoglycemic and hyperglycemic mice

**Mouse strain**	**Glycemic status**	**Stages 1-4 (% of mice)**	**Stage 5 (% of mice)**	**Latency (min)**	**Duration (min)**
FVB/NJ	Normoglycemic	100	96.37	32.8 ± 1.6	69.6 ± 4.7
C57BL/6	Normoglycemic	100	93.76	30.5 ± 1.3	76.3 ± 2.8
C57BL/6	Hypoglycemic	100	89.34	30.4 ± 6.4	55.4 ± 14.4
C57BL/6	Non-ketotic hyperglycemic	100	92.58	49.6 ± 6.1^**a**^	66.2 ± 4.0
C57BL/6	STZ-hyperglycemic	100	96.08	36.5 ± 4.6	93.3 ± 2.9^**b**^

KA induced a similar level of stage, latency and seizure duration irrespective of mouse strain. A significant difference in latency to onset of stage 4 seizures was observed in non-ketotic hyperglycemic mice as compared to normoglycemic C57BL/6 mice (^**a**^F=7.50; P=0.03) and a significant difference in duration of stage 4/5 seizures was observed in STZ-hyperglycemic mice as compared to normoglycemic C57BL/6 mice (^**b**^F=17.56; P=0.006).

### Glucose infusion restores glycemic control following KA-induced SE

**Figure 1:**
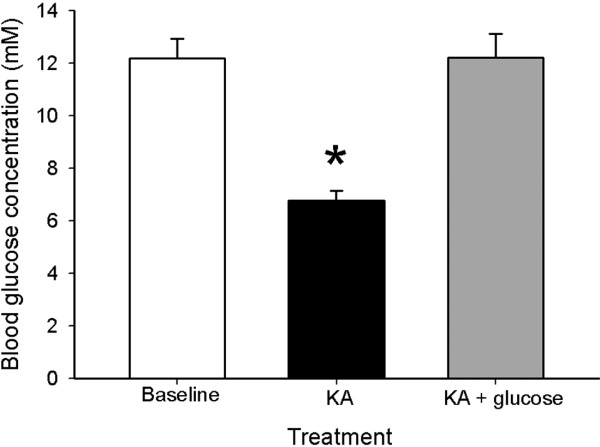
**Blood glucose concentrations following KA and following glucose treatment.** Blood glucose concentrations measured 3 hours following kainate-induced seizures and 24 hours following an injection of 1 ml of 20% glucose. Note that acute glucose treatment restored blood glucose concentrations to near baseline levels, while kainate administration resulted in a seizure-associated decrease in blood glucose concentration. Data represent the mean ± S.E.M. of at least 5 mice per condition. Asterisk indicates significant difference compared with any other group (ANOVA with post hoc Student Newman Keuls; F=23.61, P<0.001).

Subcutaneous administration of KA at a dose of 20 mg/kg of body weight into FVB mice resulted in a 2-fold reduction in blood glucose from 12 mM in vehicle-injected controls to 6 mM when measured three hours following administration (Figure 1). Interestingly, intraperitoneal injection of 20 mg/ml glucose into FVB mice once a day for 3 days following KA administration restored blood glucose levels in mice to values similar to controls (FVB mice, saline-treated), suggesting that exogenous administration of glucose can protect against glucose dysregulation following KA.

### Glucose infusion protects mice against post-seizure hippocampal pathology

**Figure 2:**
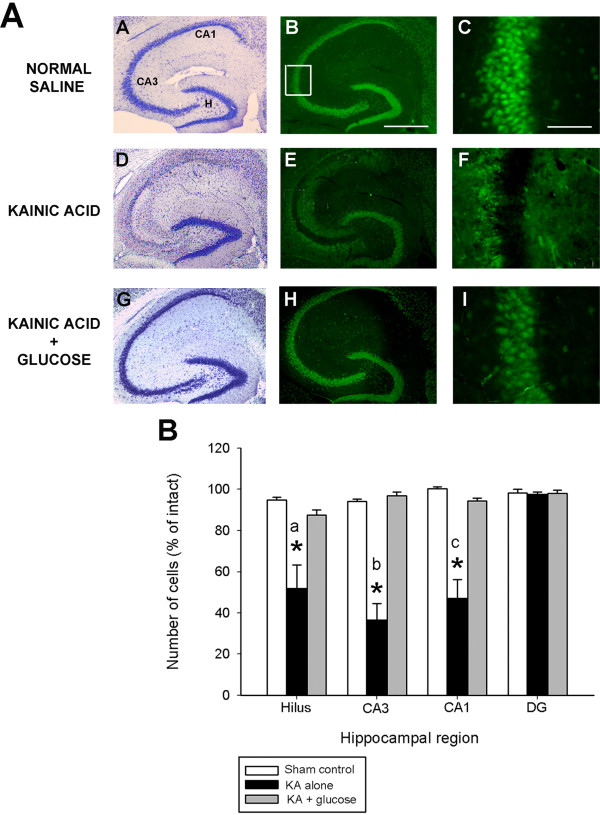
**Glucose infusion suppresses neuronal damage following kainate-induced status epilepticus.** (**A**) Low-magnification photomicrographs of cresyl violet (A,D,G) and NeuN-immunofluorescent (B,E,F) stained horizontal sections of the hippocampus illustrating surviving cells throughout the hippocampus 7 days following systemic kainate administration to FVB mice. High-magnification NeuN-immunofluorescent stained horizontal sections of area CA3 (C,F,I) among the three groups. Neuron loss following status epilepticus was profoundly reduced when exogenous glucose was administered for 3 consecutive days following seizure induction. Note the significant amount of cell loss in representative sections from KA-treated mice in the dentate hilus, area CA3 and area CA1, depicted as an absence of cresyl violet stain (D) or NeuN-immunofluorescence (E). Cell loss was not observed after post-seizure glucose infusion (G,H,I). CA1 and CA3 denote the hippocampal subfields; H, dentate hilus. Scale bar = 750 μm (A,C); 100 μm (B,D). High-magnification photomicrographs represent details of the boxed area of CA3 shown in B. (**B**) Quantitative analysis of neuronal density in hippocampal subfields seven days following KA administration to FVB mice. Viable surviving neurons were estimated by cresyl violet staining. Bars denote the percentage of surviving neurons (as compared with saline-injected sham control FVB mice). Data represent the mean ± S.E.M. of at least 5 mice per condition. Asterisks indicate significant difference compared with KA alone or Sham control of P<0.05. (ANOVA with post hoc Student Newman Keuls; a: F=9.68, P=0.003; b: F=42.87, P<0.001; c: F=23.76, P<0.001).

As shown in Figure 2A, horizontal sections from FVB mice sacrificed 7 days following kainate injection and processed for cresyl violet staining and NeuN immunofluorescence, confirmed that administration of KA led to the degeneration and loss of CA3 and CA1 pyramidal neurons and dentate hilar neurons, as evidenced by a loss of cresyl violet staining and NeuN-immunostaining (Figure 2A, panels D,E). In accordance with previous studies [30,32,41,42], cells within the dentate granule cell layer and area CA2 of Ammon’s horn were spared. In contrast, representative sections from glucose-treated mice showed a dramatic reduction in the extent of cell loss throughout all hippocampal cell fields. In particular, FVB mice administered glucose following KA-induced SE exhibited no detectable reduction of neurons within the hippocampus proper (Figure 2A, panels G,H), and no indication was noted of damage to neuronal nuclei in any hippocampal region or in the septum, amygdala, pyriform cortex, neocortex, or thalamic nuclei. Our results suggest that the protection against excitotoxic cell death by the relatively small dose of glucose results from glycemic control after KA.

In accordance with previous studies [23,27], quantitative analysis of subfield group means revealed that mice susceptible to seizure-induced cell death via KA administration (FVB) displayed a reduction of 44% dentate hilar neurons, 64% of CA3 pyramidal neurons, and 49% of CA1 pyramidal neurons seven days after KA administration (F=17.72 ; P<0.001; Figure 2B). In contrast, administration of glucose significantly reduced neuronal damage in FVB mice (F=16.38; P<0.001). Post hoc analyses revealed a dramatic protective effect of glucose following KA administration in area CA3 (p<0.001), area CA1 (p<0.001); and the dentate hilus (p< 0.001), as compared to FVB mice treated with KA alone.

### Effect of hypoglycemia on blood glucose concentration and kainate-induced seizure severity

**Figure 3:**
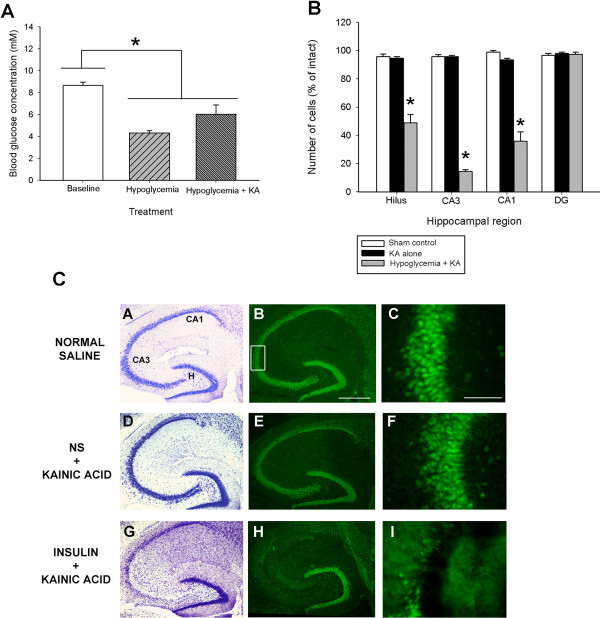
**Glucose infusion suppresses neuronal damage following kainate-induced status epilepticus damage.** (**A**) Blood glucose concentration was decreased significantly 3 hours after insulin injection and after insulin + kainate injections. Data represent the mean ± S.E.M. of at least 5 mice per condition. Asterisk indicates significant difference compared with any other group (ANOVA with post hoc Student Newman Keuls; F=16.57, P<0.001). (**B**) Quantitative analysis of neuronal density in hippocampal subfields following insulin injection + KA to B6 mice. Viable surviving neurons were estimated by cresyl violet staining. Bars denote the percentage of surviving neurons (as compared with saline-injected sham control B6 mice). Data represent the mean ± S.E.M. of at least 5 mice per condition. Asterisks indicate significant difference compared with KA alone or Sham control (ANOVA with post hoc Student Newman Keuls; F=99.70, P<0.001). (**C**) Insulin-induced hypoglycemia dramatically increased the extent of hippocampal cell death in B6 mice. Low-power photomicrographs of cresyl violet (A,D,G) and NeuN-stained (B,E,H) horizontal sections of the hippocampus showing differential cell loss 7 days after kainate-induced SE in a normoglycemic (NS + KA) and hypoglycemic (insulin + KA) mouse. Note that extensive kainate-induced cell loss, as evidenced by loss of cresyl violet staining (G) and NeuN immunostaining (H), is only observed in the dentate hilus, areas CA3 and CA1 following insulin pre-treatment in excitotoxin cell death resistant (B6) mice. CA1 and CA3 denote the hippocampal subfields; H, dentate hilus. Scale bar = 750 μm (A,C); 100 μm (B,D). High-magnification photomicrographs represent details of the boxed area of CA3 shown in B.

Our data confirms previous studies [43,44] showing that hypoglycemia (induced by bolus insulin administration) significantly modulated glucose levels. Blood glucose levels following bolus injection of insulin were significantly lower by nearly 2-fold as compared to baseline normo-glycemic levels in B6 mice (Figure 3A). As well, blood glucose levels measured 3 hours following bolus insulin administration and kainate administration were also significantly lower than corresponding values in normoglycemic B6 mice without kainate administration.

To determine if hypoglycemia could modulate seizure severity, B6 mice were treated with kainic acid and seizure severity was assessed. As shown in Table 1, we found no difference between B6 mice that were hypoglycemic versus vehicle injected mice with regard to seizure sensitivity, as evidenced by no alterations in latency to onset of first severe seizure (P=0.990) or duration of severe seizures (P=0.24). Thus, hypoglycemia was without effect on KA-induced seizure activity.

### Hypoglycemic mice show increased hippocampal neurodegeneration

Following systemic administration of KA to young adult B6 mice, essentially no cell death was observed within the hippocampus or within any other region of the brain, in accordance with previous results [23,27], and as evidenced by no loss of cresyl violet or NeuN-immunostaining (Figure 3C, panels D,E). In contrast, in B6 mice with insulin-induced hypoglycemia followed by KA-induced SE, we observed significant cell loss, as evidenced by decreased cresyl violet (Figure 3C, panel G) and NeuN-immunostaining in three hippocampal subfields (dentate hilus, area CA3 and area CA1; Figure 3C, panel H).

In particular, quantitative analysis of subfield group means revealed that hypoglycemic B6 mice that underwent KA-induced SE displayed a reduction of nearly 50% of dentate hilar neurons, over 80% of CA3 pyramidal neurons, and nearly 70% of CA1 pyramidal neurons (Figure 3B). Thus, a significant reduction in hippocampal neurons within the dentate hilus (F=64.31; P<0.001), area CA3 (F=150.33; P<0.001) and area CA1 (F=99.70; P<0.001) was observed. In contrast, normoglycemic B6 mice that underwent KA-induced SE displayed no detectable evidence of reduction of neurons in any of the hippocampal subfields after KA administration. These results demonstrate that B6, which are typically excitotoxin cell death resistant, exhibit seizure-induced cell death when they undergo insulin-induced hypoglycemia followed by KA-induced SE.

### Effects of STZ-induced diabetes and hyperglycemia on blood glucose concentration

**Figure 4:**
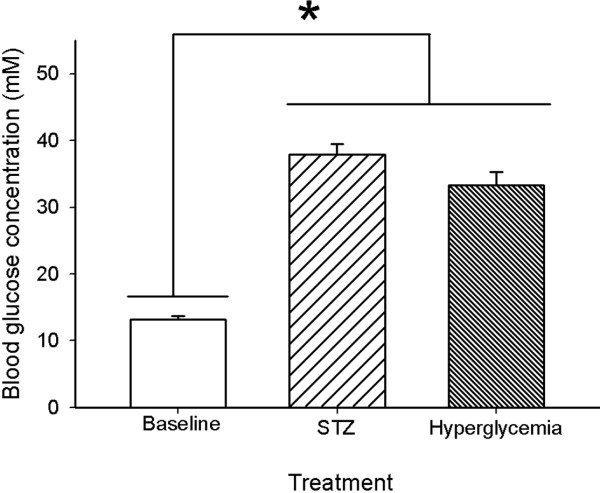
**Blood glucose concentrations following STZ and non-ketotic hyperglycemia in B6 mice.** Streptozocin (STZ) diabetic mice developed full diabetes with blood glucose concentrations greater than 30 mM. An acute glucose injection was used as a non-STZ model of hyperglycemia. Note that there was no significant difference in blood glucose concentration between the two models of hyperglycemia prior to kainate administration. Data represent the mean ± S.E.M. of at least 5 mice per condition. Asterisk indicates significant difference (ANOVA with post hoc Student Newman Keuls; F=105.54, P<0.001).

Diabetes was induced by streptozotocin (STZ), a glucosamine-nitrosourea compound that damages pancreatic ß cells, resulting in hypoinsulinemia and hyperglycemia [45]. Three weeks after the streptozotocin injection, more than 90% of mice injected with STZ developed diabetes shortly after injection with tail blood glucose concentrations higher than 25 mM . The elevated blood glucose persisted and worsened throughout the study with diabetic B6 mice having an average blood glucose of 37 ± 1.56 mM (Figure 4). Similarly, mice that received a bolus injection of 20% glucose to create a condition of nonketotic hyperglycemia independent of diabetes also displayed elevated blood glucose concentrations that were significantly different from normo-glycemic mice (Figure 4; F=195.54; P<0.001). Injections of citric acid (vehicle) produced no diabetes; glucose concentrations were 13.2 ± 0.5 mM.

### Diabetic hyperglycemia and non-ketotic hyperglycemia modulate seizure sensitivity

Previous studies have suggested that glycemic modulation can alter seizure threshold [44,46]. Thus, in the present study, we used two models of hyperglycemia to assess the effects of glycemic modulation on seizure induction and seizure duration in B6 mice. While we found no qualitative differences in seizure intensity irrespective of the model utilized to induce hyperglycemia (no differences in the percentage of mice achieving status epilepticus, Table 1), we did find differences between the models with regard to latency to onset of stage 4 seizures (Table 1). In particular, we found a significant increase in latency to onset of stage 4 seizures in our model of hyperglycemia as compared to vehicle-injected animals (KA=30.50 ± 1.32 mins vs. Hyperglycemia + KA= 49.60 ± 6.06 mins, P=0.03), but no significant difference in latency when we compared STZ-induced hyperglycemia vs. vehicle alone (P=0.25). As well, we found significant differences in the duration of stage 4 seizures depending on our model of induction of hyperglycemia. While we found no significant differences in seizure duration between hyperglycemic versus vehicle-injected mice (P=0.09), we did find a significant difference in seizure duration when comparing STZ-induced hyperglycemic mice versus controls (KA=76.25 ± 2.83 vs. STZ + KA= 93.25 ± 2.90, P=0.006).

### Diabetic hyperglycemia aggravates the extent of seizure-induced cell death in C57BL/6 mice post-kainate administration

**Figure 5:**
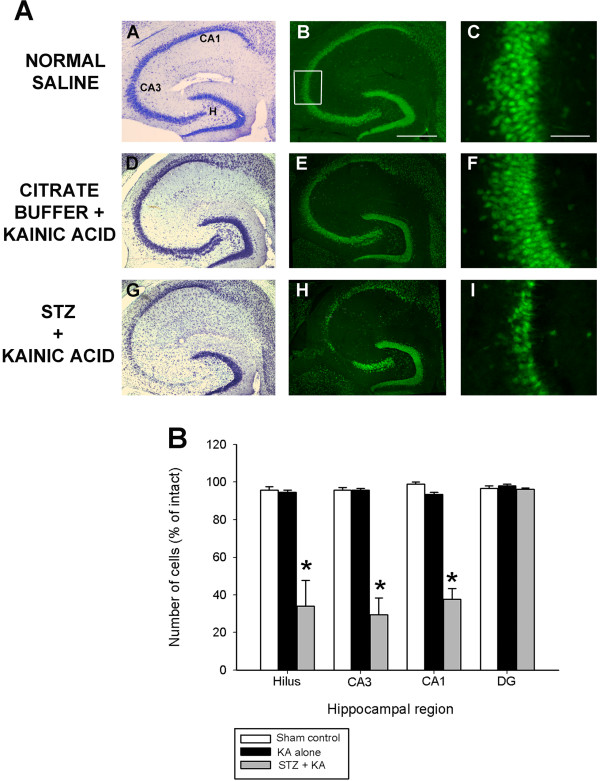
**Diabetic-induced hyperglycemia aggravates status epilepticus-induced hippocampal damage.** (**A**) Low-power photomicrographs of cresyl violet (A,D,G) and NeuN-immunofluorescent-stained (B,E,H) horizontal sections of the hippocampus illustrating surviving cells throughout the hippocampus 7 days following systemic kainate administration to vehicle-treated, and STZ-treated mice that underwent KA-induced SE. STZ-induced hyperglycemia dramatically increased the extent of hippocampal cell death in excitotoxin cell death resistant B6 mice. Note the significant amount of cell damage in representative sections from STZ + KA treated mice as evidenced by a loss of cresyl violet staining (G) and NeuN-immunofluorescence (H) and complete absence of any seizure-associated cell loss in KA treated mice (D,E). CA1 and CA3 denote the hippocampal subfields; H, dentate hilus. Scale bar = 750 μm (A,C); 100 μm (B,D). High-magnification photomicrographs represent details of the boxed area of CA3 shown in B. (**B**) Quantitative analysis of neuronal density in hippocampal subfields seven days following STZ + KA administration to B6 mice. Viable surviving neurons were estimated by cresyl violet staining. Bars denote the percentage of surviving neurons (as compared with saline-injected sham control B6 mice). Data represent the mean ± S.E.M. of at least 5 mice per condition. Asterisks indicate significant difference compared with KA alone or Sham control of P<0.05. (ANOVA with post hoc Student Newman Keuls; F=195.54, P<0.001).

Streptozotocin (STZ) is a ß-cytotoxic agent widely used to induce diabetes in rodents [47,48]. The STZ model has been used extensively in studies in the patholophysiology of diabetes and its complications [49,50]. We examined differences in status-induced neuronal injury in B6 mice treated with vehicle and kainate alone with those that exhibited STZ-induced diabetes. As shown in Figure 5A, we found significant differences in the extent of injury between these two groups when cell death was measured 7 days later. Consistent with our previous studies [23,27], irrespective of the hippocampal area examined, administration of KA to nearly excitotoxin-resistant mice (B6) resulted in no detectable reduction in hippocampal neurons within any subfields of the hippocampus proper (Figure 5A, panels D,E,F). In contrast, administration of KA to STZ-induced diabetic mice led to the profound neuronal loss of dentate hilar, area CA3 and area CA1 cells, as evidenced by a loss of cresyl violet staining (Figure 5A, panel G) and NeuN-immunofluorescence (Figure 5A, panels H,I).

Quantitative analysis of hippocampal subfield group means (Figure 5B) revealed a significant reduction in neuronal cell loss within the dentate hilus (F=23.28; P<0.001), area CA3 (F=64.73; P<0.001) and area CA1 (F=112.57; P<0.001) of diabetic hyperglycemic mice that underwent KA-induced SE. Thus, we found that compared with non-diabetic mice, diabetic mice lost more hippocampal neurons during the acute stage after status epilepticus. In contrast, we observed no significant cell loss in B6 mice that were injected with vehicle prior to KA-induced SE. These results demonstrate that B6, which are typically excitotoxin cell death resistant, exhibit seizure-induced cell death when they undergo diabetes-induced hyperglycemia followed by KA-induced SE.

### Non-ketotic hyperglycemia increases the extent of seizure-induced cell death in C57BL/6 mice post-kainate administration

**Figure 6:**
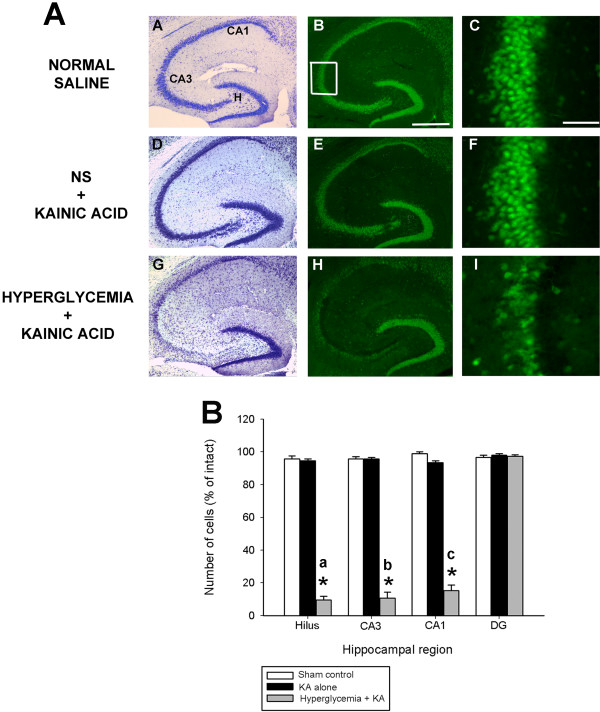
**Hyperglycemia aggravates status epilepticus-induced hippocampal damage.** (**A**) Low-power photomicrographs of cresyl violet (A,D,G) and NeuN immunofluorescent-stained (B,E,H) horizontal sections of the hippocampus illustrating surviving cells throughout the hippocampus 7 days following systemic kainate administration to normoglycemic and non-ketotic hyperglycemic mice that underwent KA-induced SE. Hyperglycemia dramatically increased the extent of hippocampal cell death in excitotoxin cell death resistant B6 mice. Note the significant amount of cell loss, as evidenced by a loss of cresyl violet staining (G) and NeuN immunofluorescence (H), in representative sections from hyperglycemic KA-treated mice throughout all excitotoxin cell death susceptible regions (G,H,I) and absence of cell loss in KA-treated excitotoxin cell death resistant mice (D,E). CA1 and CA3 denote the hippocampal subfields; H, dentate hilus. Scale bar = 750 μm (A,C); 100 μm (B,D). High-magnification photomicrographs represent details of the boxed area of CA3 shown in B. (**B**) Quantitative analysis of neuronal density in hippocampal subfields following hyperglycemia + KA to B6 mice. Viable surviving neurons were estimated by cresyl violet staining. Bars denote the percentage of surviving neurons (as compared with saline-injected sham control B6 mice). Asterisks indicate significant difference compared with KA alone or Sham control of P<0.05. (ANOVA with post hoc Student Newman Keuls; a: F=781.34; P<0.001; b: F=542.49; P<0.001; c: F=567.89; P<0.001).

As shown in Figure 6A, systemic administration of KA to normoglycemic B6 mice was without effect on the induction of any cell loss throughout any hippocampal subfields (panels D,E,F). C57BL/6 mice showed no detectable reduction of neurons in the following hippocampal areas: dentate hilus, area CA3, area CA1, and the dentate granule cell layer; nor to neuronal nuclei in the septum, amygdala, pyriform cortex, neocortex or thalamic nuclei. These results are in accordance with previously obtained results [23,27]. In contrast, diabetic mice that underwent KA-induced SE displayed a reduction of hippocampal neurons in areas CA3, and CA1 and within the dentate hilus, as seen by a loss of cresyl violet staining (Figure 6A, panel G) or NeuN immunofluorescence (Figure 6A, panels H,I).

Quantitative analyses of hippocampal neuron numbers showed a dramatic and significant reduction on average of 90% of dentate hilar neurons (F=781.34; P<0.001), CA3 pyramidal neurons (F=542.49; P<0.001), and CA1 pyramidal neurons (F=567.89; P<0.001) 7 days after KA administration as compared with normoglycemic mice (Figure 6B). These results indicate that glycemic modulation can induce differential vulnerability of neurons in the hippocampus.

## Discussion

The effects of glycemic modulation on excitotoxic cell death and seizure susceptibility are diverse and complex. It has previously been established that limited energy availability compromises hippocampal neuronal viability during status epilepticus [51-53]. As seizure-induced excitotoxic cell death is likely due to excessive glutamate release and excessive cytosolic calcium [54], these can be worsened by energy failure. In particular, intracellular sequestering of Ca^2+^ and the concomitant transmembrane extrusion of Ca^2+^ are directly or indirectly ATP-dependent [55], and can result in impaired ATP production [56], the release of reactive oxygen species [57], and the subsequent release of proteins involved in the cell death cascade [58-61]. As a result, many of the steps mediating seizure-induced excitotoxic cell death are sensitive to energy availability. In the present study, we examined the effects of glycemic modulation on excitotoxic cell death and seizure susceptibility following systemic administration of the chemoconvulsant, kainic acid to inbred strains of mice. We demonstrated that injection of glucose following KA-induced SE was profoundly neuroprotective against seizure-induced neuronal damage. As well, we also found that compared with normoglycemic mice, (1) hyperglycemic were less seizure sensitive, (2) diabetic hyperglycemic mice were more seizure sensitive, and (3) hypo-, hyper- or STZ-hyperglycemic mice showed increased susceptibility to seizure-induced cell death after status epilepticus.

### Glycemic control is neuroprotective in CNS injury

In order to assess the putative ‘neuroprotective’ effects of controlling glycemic status in mice that underwent KA-induced SE, we administered glucose 3 hours following KA-induced status epilepticus and then gave two additional injections 24 and 48 hours following the initial glucose injection. We report that FVB mice are hypoglycemic following KA-induced SE and that glucose treatment reverses the hypoglycemic status as well as provides protection against seizure-induced cell death. Our results are in agreement with previous clinical and experimental studies on ischemic brain injury [62]. Preclinical data from animal models indicates that insulin may reduce damage in both global and focal ischemia [62,63] and in transient global ischemia, insulin has a direct neuroprotective effect on CNS parenchyma [64]. Moreover, Nagamizo et al. [65] demonstrated that a relatively small dose of preischemic insulin protects against ischemic spinal cord injury, and that the protective effect was cancelled by a concomitant glucose infusion. The idea of brain protection by insulin is not new; however, the mechanisms underlying glucose levels and altered neuronal excitability remain incompletely understood.

### Seizure susceptibility and glycemic control

As previous studies have established that there is a biphasic dependence of seizure susceptibility on blood glucose concentrations, we examined several seizure parameters (seizure intensity, seizure latency and seizure duration) in hypoglycemic and hyperglycemic B6 mice. While we found no difference between B6 mice that were hypoglycemic versus vehicle-injected mice with regard to seizure sensitivity, latency to onset of first severe seizure or duration of severe seizures, we did observe that hyperglycemia can modulate seizure susceptibility. In particular, while we found no qualitative differences in seizure intensity irrespective of the model utilized to induce hyperglycemia, we did find differences between the models with regard to latency and duration.

Interestingly, hyperglycemic mice that underwent KA-induced SE demonstrated a significant increase in the latency to onset of severe seizures, suggesting a reduction in seizure severity. In contrast, STZ mice displayed a significant increase in seizure duration following KA-induced SE, indicative of an increase in seizure sensitivity. Thus, depending on the model of hyperglycemia utilized, B6 mice appeared to be either less seizure sensitive (hyperglycemia) or more seizure sensitive (STZ-induced hyperglycemia). While no studies to date have compared seizure susceptibility differences based on the model of hyperglycemia, previous studies have found that STZ-induced diabetic rats that underwent lithium-pilocarpine induced SE had a higher seizure susceptibility than normoglycemic rats [66].

It is somewhat surprising that we saw differential effects on seizure susceptibility depending on the model of hyperglycemia utilized in our studies, as others have demonstrated that seizure susceptibility has been shown to increase with incremental blood glucose, either during experimental diabetes or acute hyperglycemia [38,67]. One potential reason for the discrepancy between our models of hyperglycemia could be the result that we compared a bolus injection of glucose to create a condition of nonketotic hyperglycemia independent of diabetes versus the STZ-induced model of type 1 diabetes (chemical ablation of the pancreatic ß cells; [68]). The underlying mechanism for the difference in seizure susceptibility between the high glucose model and STZ model is uncertain, although the high glucose model has been described as an acute hyperglycemia model, while the STZ model is described as a chronic or sustained hyperglycemia model.

### Hypoglycemia mice show increased KA-induced hippocampal degeneration

Previous studies in our laboratory have demonstrated robust strain differences with respect to susceptibility to excitatory amino acid-induced cell death [26-28]. Although C57BL/6 (B6) and FVB/N (FVB) mouse strains exhibit comparable seizure activity following systemic administration of kainic acid, FVB mice have been reported to be vulnerable to excitotoxic insults, while B6 mice are resistant to excitotoxic cell death. While the molecular and cellular events responsible for the selective vulnerability of hippocampal neurons to kainic acid are not yet fully understood, excitotoxicity is thought to be triggered by the activation of ionotropic glutamate receptors resulting in calcium dysregulation [22,54,69], oxidative stress and mitochondrial dysfunction, and the initiation of signaling cascades within susceptible neurons resulting in cell death [70,71].

Our results demonstrate that hypoglycemia induced a phenotypic switch in B6 mice, previously characterized as resistant to seizure-induced excitotoxic cell death. In particular, B6 (excitotoxin cell death resistant) mice with insulin-induced hypoglycemia following by KA-induced SE exhibited greater seizure-induced cell death. We are not aware of any studies that compared the effects of hypoglycemia on status epilepticus associated injury. However, our results suggest that hypoglycemia aggravates excitotoxic neuronal death in a nearly excitotoxin-resistant brain.

The mechanism of hypoglycemia-induced injury is thought to be the result of several contributing factors acting downstream of glucose deprivation, and not just a loss of energy supply (e.g. glucose) from neurons [72]. One of these contributing factors includes the sustained activation of glutamate receptors [73], as well as increased mitochondrial membrane permeability [74]. Differences in the number and function of neurotransmitter receptors, molecular and biochemical pathways of energy metabolism and/or susceptibility to respiratory depression may also be responsible for the increased vulnerability to excitotoxic cell death. At present, while we cannot explain why induction of hypoglycemia prior to KA-induced SE renders mice previously characterized as excitotoxin cell death resistant to become susceptible; it is possible that excessive glutamate activation (via acute hypoglycemia combined with stimulation of ionotropic glutamate receptors via kainate administration) drives glutamate release to excessive levels that cannot be resolved when only one insult is presented. Additional studies must clarify the specific cellular mechanisms that promote neurotoxicity in C57BL/6 mice.

### Hyperglycemia aggravates the extent of KA-induced cell death

In the present study, we found that STZ-induced and glucose-induced hyperglycemia exacerbated the neuropathological consequences of KA-induced SE in excitotoxin-resistant mice. Thus, both acute and chronic hyperglycemia produced the same outcome of increased susceptibility to seizure-induced excitotoxic cell death. Our results demonstrating that both models of hyperglycemia rendered hippocampal neurons more vulnerable to KA-induced excitotoxic cell death are in agreement with previous clinical and animal studies. In particular, previous studies have demonstrated that status epilepticus and its associated neuropathological consequences is not an uncommon complication associated with diabetic hyperglycemia [33,35]. Diabetes has been suggested to exacerbate status epilepticus-induced brain damage, result in poor recovery following status, and even increase mortality [38,75,76]. Moreover, animal studies have demonstrated that high glucose concentrations are associated with an increased susceptibility to seizures and augmented brain damage in the pilocarpine model of temporal lobe epilepsy [66]. Hyperglycemia is known to modify many proteins important for cell survival by advanced glycation, inducing some death-related proteins like high mobility group box 1, which triggers the expression of pro-inflammatory mediators, or downregulation of cytoskeletal proteins that support neuronal cell survival [77,78]. Thus, the prolonged effects of hyperglycemia may be the result of an additive effect of toxicity on the brain.

## Conclusion

In summary, we found that glycemic control could rescue hippocampal cells from seizure-induced excitotoxic cell death in an excitotoxin-susceptible mouse strain, FVB. As well, the results presented here illustrate that hyper- or hypoglycemia additively increased the extent of seizure-induced cell death in an excitotoxin-resistant mouse strain, B6. The ability of glucose dysregulation to elicit a phenotypic switch from excitotoxin resistant to susceptible after kainate administration implicates glucose dysfunction as a key event in the pathogenesis of seizure-induced excitotoxic cell death. While the specific pathophysiological mechanisms underlying this relationship remain unclear, our data support the hypothesis that deficient insulin signaling may represent a critical contributing factor in the susceptibility to seizure-induced cell death and this may be an important therapeutic target. An understanding of the interplay between glucose regulation and excitotoxic neurodegeneration will have important consequences, not only in the context of epilepsy, but for hypoxia, stroke, and other related pathologies.

## Methods

All experiments were conducted in accordance to the guidelines set forth by the National Institutes of Health. All procedures were approved by the University of Southern California Institutional Animal Care and Use Committee. Animals were housed under controlled conditions (12 hour light/12 hour dark), and food and water were provided to the mice *ad libitum*.

### Animals

Four separate cohorts of mice were used for these studies. Six to eight-week old FVB/N male mice (Jackson Laboratories, Bar Harbor, ME), housed in groups of 4–5 per cage, were used for the KA + exogenous glucose experiments. Three separate cohorts of six to eight-week old C57BL/6 (Jackson Laboratories, Bar Harbor, ME), housed in groups of 4–5 per cage, were used for the hypoglycemia + KA, hyperglycemia + KA, and Streptozotocin (STZ) + KA experiments. *All of the procedures used in these experiments were in accordance with the NIH Guide and approved by the USC Animal Care and Use Committee.* All efforts were made to minimize the number and suffering of any animals used in these experiments.

### Induction of hypoglycemia

On the experimental day, mice were weighed and the baseline blood glucose was measured. Mice were injected i.p. with 1 I.U./kg of regular human insulin (Humalin, Eli Lilly, Indianopolis, IN), freshly dissolved in normal saline. Blood glucose from blood was measured prior to insulin administration (basal glucose concentration), and at 2 additional timepoints. Blood glucose was measured 30 minutes following insulin administration, prior to kainate administration and 3 hours following kainate administration. Mixed peripheral blood (arterial, venous, and capillary) samples were obtained after snipping the tip of the tail. A droplet of blood (~2 μl) was collected on the test strip and evaluated using a One-Touch Glucosemeter (LifeScan, Millipitas, CA). As the sample size of the blood droplet was minimal, there was no adverse effect to the well being of the mouse and only a single cut of the tip of the tail was needed to provide all samples. Blood glucose levels of <5.1 mM were defined as hypoglycemic.

### Induction of hyperglycemia

One group of mice from each strain was fasted overnight and intraperitoneally injected with 1 ml of a 20% glucose solution 30 minutes prior to KA-induced SE to create a condition of nonketotic hyperglycemia independent of diabetes [38]. Blood glucose from blood was measured prior to glucose administration (basal glucose concentration) and 3 hours following kainate administration.

A second group of mice was fasted overnight and administered 200 mg/kg, i.p. streptozotocin (STZ; Sigma Aldrich, St. Louis, MO) in 100 mM citrate buffer (pH 4.5), an antibiotic that destroys the insulin-secreting ß cells of the pancreas [47,48] and has previously been used to induce chronic hypoinsulinemia in rodents [49,50]. Controls received citrate buffer alone. Both sets of mice underwent kainate-induced status epilepticus three weeks following drug administration. Blood glucose was measured prior to STZ administration (basal glucose concentration), and monitored weekly following STZ administration (for a total of 3 weeks) in peripheral blood obtained after snipping the tail. A droplet of blood (~2 μl) was collected on the test strip and evaluated using a One-Touch Glucosemeter (LifeScan, Millipitas, CA). Mice with blood glucose values > 250 mg/dl were included in the STZ group.

### Kainate-induced status epilepticus

Sustained seizures (status epilepticus) were induced in animals by the administration of kainic acid (KA), a potent agonist of the AMPA/KA class of glutamate receptors. KA was dissolved in isotonic saline (pH 7.4) and administered subcutaneously to adult mice at a dose of 20 mg/kg (Nanocs, New York, NY). Following KA administration, mice were monitored continuously for 4 h for the onset of locomotor activity and behavioral manifestations of limbic seizure episodes, as described previously [23-26,28]. Status epilepticus was defined as continuous behavioral seizure activity lasting at least 1 hour or a series of intermittent seizures without restoration of normal behavioral patterns between successive seizures. Mice were scored for seizure activity using a previously defined six-point seizure scoring scale [27] that was adapted from a five-point scale for rats [79].

Seizure stages were defined as follows: Stage 1, immobility; Stage 2, forelimb and/or tail extension, rigid posture; Stage 3, repetitive movements, head bobbing; Stage 4, rearing and falling; Stage 5, continuous rearing and falling; and Stage 6, severe tonic-clonic seizures. Only those mice exhibiting at least 45 min of continuous stage 4/5 seizures were included in this study, as previous studies have suggested that there is a direct relationship between the generation of epileptiform activity and the extent of damage in hippocampal subfields [18,32,80]. Seizure parameters monitored included latency of convulsions and duration of severe (Stage 4/5) seizure activity. All experiments were approved by the Institutional Animal Care and Use Committee (IACUC) of the University of Southern California and conducted in accordance with its guidelines. Every effort was made to minimize animal suffering and to minimize the number of animals utilized in order to produce reliable scientific data.

### Administration of 20% glucose after KA-induced SE

Administration of 1 ml of 20 mg/ml glucose (intraperitoneal) was initiated 3 hours following KA-induced status epilepticus and two additional injections were given 24 and 48 hours following the initial glucose injection. Blood glucose from blood was measured prior to glucose administration and 30 minutes following each glucose injection.

### Tissue preparation and histology

In order to evaluate the severity of kainate-induced excitotoxic brain damage, brains from each strain of mice were processed for light microscopic histopathologic evaluation according to previously published methods [29]. Briefly, 7 days after seizure induction by kainate, mice were anesthetized with Avertin and transcardially perfused with 4% paraformaldehyde in 0.1M phosphate buffer (pH 7.4). Brains were removed and post-fixed overnight, followed by cryoprotection in 30% sucrose for at least 12–18 h. Horizontal (40 μm) frozen sections were cut on a sliding microtome and collected as free-floating sections in 0.1M phosphate buffer (pH 7.4) until processed for light microscopic histology. Every sixth section (~240 μm) was processed for cresyl violet staining to assess cell loss and morphology.

### NeuN immunofluorescence

Immunofluorescence was performed on an additional series of sections (every sixth section; ~240 μm) to detect those neurons that survived 7 days following kainate-induced SE. For immunofluorescent labeling, sections were washed with 0.1M phosphate buffer (pH 7.4) and blocked with 5% normal serum and 0.1% Triton X-100 in 0.1M phosphate buffer (pH 7.4). Next, sections were incubated overnight with a neuronal marker against NeuN (monoclonal from mouse; Millipore, Billarica, MA; 1:500) at 4°C. After several washes, sections were incubated with a secondary antibody from mouse conjugated with Cy2 (1:200; Jackson ImmunoResearch, West Grove, PA) for 2 h at room temperature. After rinsing, sections were mounted and coverslipped with ProLong anti-fade mounting medium (Molecular Probes, Eugene, OR). For labeling, omission of the primary antibody served as negative control. Labeling for NeuN was viewed under an Olympus BX51 fluorescence microscope (Olympus, New York, NY).

### Quantitative analysis of hippocampal cell loss

Subsequently, to determine the susceptibility of individual hippocampal subfields to neurotoxic insult, we counted neurons in Nissl-stained sections. Quantitative analysis of hippocampal cell loss was performed by an observer blinded to the strain groups using unbiased stereological methods on cresyl violet-stained sections according to previously published protocols [23,27,28]. The Nissl-stained neurons in area CA3, area CA1, the dentate hilus, and the dentate gyrus were counted in both the right and left hippocampus and counting was initiated within the ventral hippocampus at the first point where hippocampal subfields could be easily identified. This level corresponded to horizontal section 54, based on the atlas of Sidman et al. [81]. Hippocampal subfields were based on Franklin and Paxinos [82] classification and discrimination between the CA3 and the dentate hilus region was based on morphological features and locations of the cells [83,84]. Specifically, for dentate hilar cell counts, the hilus was operationally defined as the region bordered by the supra- and infrapyramidal granule cell layers and excluding the densely packed pyramidal neurons of area CA3.

Neuron counts were made in all subfields and the numbers for each side were averaged into single values for each animal. Surviving cells were counted only if they were contained within the pyramidal cell layer, dentate hilus or dentate gyrus, possessed a visible nucleus and characteristic neuronal morphology and had a cell body larger than 10 μm. Six square counting frames (200 X 200 μm) were randomly placed in the pyramidal layer of fields CA1 and CA3 or in the dentate gyrus in 4–5 regularly spaced horizontal sections from each animal. Two square counting frames were randomly placed in the dentate hilus in 4–5 regularly spaced horizontal sections from each animal. Neuronal nuclei were evaluated at three different focal planes and only those in the focal plane were counted with a 40X objective and considered as a counting unit. Stereological analysis was performed with the aid of ImagePro Plus 4.5 software (Media Cybernetics, Silver Spring, MD) and a motorized Z-stage (Optiscan; Prior Scientific, Fairfax, VA). Final cell counts were expressed as the percentage of cells as compared to intact mice. Results were assessed statistically by one-way analysis of variance (ANOVA) using the computer program, SigmaStat (Jandel Scientific, San Rafael, CA), and intergroup differences were analyzed by Newman-Keuls post hoc test.

## Abbreviations

ANOVA, Analysis of variance; C57BL/6, B6; CNS, Central nervous system; FVB/N, FVB; KA, Kainic acid; KA-SE, Kainate-induced status epilepticus; SE, Status epilepticus; STZ, Streptozotocin; TLE, Temporal lobe epilepsy.

## Authors’ contributions

PES conceived of the study, carried out the experiments, interpreted the data, and drafted the manuscript. All authors read and approved the final manuscript.
